# Comparative Preclinical Analysis of Anti-B7-H3 CAR-T Cells Targeting Neuroblastoma

**DOI:** 10.3390/biomedicines13092130

**Published:** 2025-08-31

**Authors:** Dzmitry V. Lutskovich, Alexander N. Meleshko, Valeria M. Stepanova, Dmitri O. Dormeshkin, Yury P. Rubtsov

**Affiliations:** 1 Belarusian Research Center for Pediatric Oncology, Hematology and Immunology, 223053 Minsk, Belarus; lutskovichdm@gmail.com; 2 Shemyakin-Ovchinnikov Institute, Bioorganic Chemistry of the Russian Academy of Sciences, Moscow 117997, Russia; ukrainskaya49@gmail.com (V.M.S.); yrubtsov@ibch.ru (Y.P.R.); 3 Institute of Bioorganic Chemistry of the National Academy of Sciences of Belarus, 220141 Minsk, Belarus; dormeshkin@gmail.com

**Keywords:** neuroblastoma, chimeric antigen receptor (CAR), B7-H3, solid tumor, cancer target, CAR-T cell functional activity, preclinical study

## Abstract

**Background**: Neuroblastoma is a childhood tumor that is usually fatal after relapse in high-risk patients. Most clinical trials of CAR-T therapy for neuroblastoma are based on targeting the disialoganglioside GD2. B7-H3, a protein from the immunoglobulin superfamily, is a specific marker for neuroblastoma and a number of other solid tumors. We conducted a preclinical study of three variants of anti-B7-H3 CAR-T cells in order to justify the selection of the best candidate for subsequent clinical trials. **Methods**: The expression level of B7-H3 was measured in a number of cell lines and neuroblastoma tissue samples via flow cytometry. The functional activity of CAR-T cells was compared using an NFAT-inducible reporter assay, a cytotoxicity test, cytokine production, and a repeated stimulation assay. **Results**: The obtained CAR-T cells carrying all resulting CAR variants specifically recognized and killed B7-H3-positive tumor cells in vitro. Nevertheless, TE9-28z and 8H9-28BBz demonstrated superior activation and cytokine production compared to the second-generation 8H9-BBz construct. TE9-28z and 8H9-28BBz exhibited functional differences in expansion, exhaustion markers, and cytokine secretion in co-cultures with target cells in vitro. In particular, TE9-28z induced higher IFNγ production, while 8H9-28BBz showed increased TNFα release. Despite comparable cytotoxicity, TE9-28z and 8H9-28BBz CAR-T cells exhibited varying persistence depending on the tumor type, and showed signs of functional exhaustion upon prolonged exposure to the target antigen. **Conclusion**: TE9-28z and 8H9-28BBz were selected for further preclinical development as promising candidates for the effective targeting of B7-H3-expressing malignancies.

## 1. Introduction

Neuroblastoma (NB) is the most common pediatric extracranial solid tumor. It accounts for 8–10% of all pediatric tumors, with an incidence rate reaching approximately 1 per 100,000 children under the age of 14 [1]. Despite the intensive treatment, which commonly includes surgery, high-dose chemotherapy, and radiotherapy, the 5-year event-free survival rate does not exceed 40–45% [2]. Recently, immunotherapy has been added to the arsenal of neuroblastoma treatments available.

The most promising direction in tumor immunotherapy is adoptive T cell therapy utilizing T cells engineered to express either transgenic T cell receptors (TCR) or chimeric antigen receptors (CAR). Among these, CAR-T cell therapy has emerged as a powerful strategy for targeting cancer cells. These modified T cells can specifically recognize the surface antigens present on tumor cells. Upon recognizing and binding tumor antigens, CAR-T cells become activated and eliminate tumor cells primarily by releasing cytotoxic molecules such as granzymes, which in turn trigger cancer cell death. In clinical trials, CD19-targeted CAR-T cells have proven to be highly efficient in treating hematologic malignancies. In 2017, the FDA approved the first CD19-directed CAR-T cell therapy as a novel treatment option for patients with non-Hodgkin lymphoma and chemotherapy-refractory or relapsed acute lymphoblastic leukemia [3]. While demonstrating high efficacy in treating certain blood cancers, CAR-T cell therapy faces substantial obstacles in targeting solid tumors. The therapeutic efficiency of CAR-T cells in solid tumors can be limited by several factors, including the limited availability of tumor-specific antigens, a dense extracellular matrix that restricts lymphocyte migration, or an immunosuppressive tumor microenvironment that impairs CAR-T cell persistence and promotes their exhaustion. Identifying tumor-associated targets that are broadly expressed in both primary and metastatic lesions of solid tumors, as well as designing the optimal CAR structure, remains a key challenge.

According to the currently available early clinical trial data, disialoganglioside GD2 is the most commonly targeted antigen in CAR-T therapy for neuroblastoma [3]. Potential candidates for CAR-T therapy for neuroblastoma and other solid tumors include several targets, such as L1-CAM, tumor-associated glypicans (GPC2, GPC3), and B7-H3 [4]. B7-H3 (CD276) is a type I transmembrane glycoprotein belonging to the B7 family of immune checkpoint molecules. In humans, three isoforms of B7-H3 have been identified: two membrane-bound forms, 2Ig B7-H3 and 4Ig B7-H3, and a secreted form, sB7-H3. These isoforms have a different number of immunoglobulin-like domains generated via alternative splicing [5]. B7-H3 exerts significant immunosuppressive effects that inhibit T cell proliferation, activation, and function. B7-H3 can reduce interferon type I (IFN) production by T cells and impair the cytotoxic activity of natural killer (NK) cells [6]. B7-H3 expression is upregulated in various human malignancies, including prostate cancer, breast cancer, neuroblastoma, glioma, colorectal cancer, and pancreatic cancer, whereas its expression in normal human tissues is typically low or undetectable [7,8]. Furthermore, accumulating evidence suggests that B7-H3 plays a significant role in tumor progression, including metastasis and immune escape, and its expression correlates with poor patient prognosis and clinical outcomes.

A number of therapeutic strategies targeting B7-H3 are currently under preclinical or clinical investigation. These include bispecific antibodies, antibody–drug conjugates, agents mediating antibody-dependent cellular cytotoxicity (ADCC), and CAR-T cell therapies [9,10]. Several variants of anti-B7H3 CAR designs have been developed and tested. In preclinical trials, R.G. Majzner developed a second-generation receptor comprising scFv from the MGA271 antibody, the CD8a hinge-transmembrane domain, and the 4-1BB and CD3z signaling domains [8]. This receptor variant was modified based on research by N.A. Vitanza, which showed improved activity of this antibody with a medium-length extracellular spacer, IgG4-hinge-CH3 [11]. This second-generation MGA271-BBz receptor was translated into a clinical trial for glioma [12] and other solid tumors [13].

Xin Tang published a preclinical study of anti-B7-H3 CAR for the treatment of glioblastoma, including scFv from the 8H9 antibody, the CD8a hinge-transmembrane domain, and the CD28/CD3z signaling domains [14]. The same receptor structure was tested in vitro by S. Li on prostate cancer cell lines [15]. An alternative version of the second-generation 8H9-28H.TM-BBz receptor was tested on a preclinical model of non-small cell lung cancer [16]. The novel murine antibody, TE9, described by Birley et al., was shown to outperform other anti-B7-H3 antibodies, such as MGA271 and 376.96, when expressed in a second-generation CAR format (TE9-28z) [17]. The latest study is the only one that compared several anti-B7-H3 CAR variants in a single study. However, no direct comparison between the leader from K. Birley’s study and the 8H9 antibody-based receptor variants has been conducted previously. Data on third-generation anti-B7-H3 receptors have also not yet been published. To address these challenges, we conducted a comparative preclinical study and evaluated different variants of the anti-B7-H3 CAR. These constructs were based on two distinct ectodomains, 8H9 and TE9, recognizing different epitopes on the B7-H3 molecule. They were incorporated into either second- or third-generation CAR backbones. We systematically compared the functional activity of the resulting CAR-T cell products, including their cytotoxic potential, cytokine production, and in vitro proliferation capacity.

## 2. Materials and Methods

### 2.1. Cell Lines

The following immortalized cell lines were used in the study: T lymphocytes: Jurkat; B lymphocytes: Daudi and IM-9; monocyte cell line THP1; HEK293T cells; human neuroblastoma cell lines: IMR-32, LAN-1, and SK-N-BE(2); human osteosarcoma cell lines: 143B KHOS-240S; and a GD2+ mouse neuroblastoma cell line—NXS2. The origin of the cell lines is given in the Supplementary Table S1. The identity of all cell lines was confirmed via STR polymorphism genotyping. Mycoplasma contamination was monitored using PCR-based assays.

### 2.2. B7-H3-Positive Jurkat Cell Production

The sequence of the human *Ig4 B7-H3* gene was amplified from cDNA of the IMR-32 cell line. BamHI and EcoRI restriction sites were added to the encoding part of the gene by extending the 5′-ends of primers, preserving the Kozak sequence and the stop codon. Using these restriction sites, the PCR product was cloned into the pUltra vector (Addgene plasmid #24129, Addgene, Watertown, MA, USA). Jurkat B7-H3+ cells were obtained via lentiviral transduction and enriched via immunomagnetic selection using the EasySep™ PE Positive Selection Kit II (STEMCELL Technologies Inc., Vancouver, BC, Canada) following anti-B7H3-PE mAb staining, with a purity exceeding 99.9%.

### 2.3. Tumor Biopsy Preparation

The tumor biopsies for immunophenotyping and antigen expression analysis were obtained from the patients diagnosed with neuroblastoma or sarcoma with their informed consent and the institutional ethics committee approval provided by the Belarusian Research Center for Pediatric Oncology, Hematology and Immunology (protocol #02/16 on 20 January 2016). Tumors were minced with scissors and then strained through a 70 µm nylon cell strainer. Red blood cells were lysed in the RBC lysis buffer for five minutes at room temperature. Tumor cells were then washed, counted, and resuspended in PBS. Cell suspension was divided into 3 tubes (each one containing at least 300,000 cells) and stained with the following antibody panels: Tube 1: Control—CD45-KrO; Tube 2: CD45-KrO, CD56 FITC, CD276 PE, GD2 PB-450, CD81-APC; Tube 3: CD99-FITC, CD171-PE, CD276-PB450, CD81-APC. A complete list of antibodies used in this study is provided in Supplementary Table S2. Dead cells were excluded from analysis using 7-AAD viability stain (Thermo Fisher Scientific Inc., Waltham, MA, USA). Stained samples were run on the Beckman DxFLEX cytometer (Beckman Coulter, Brea, CA, USA) and analyzed with CytExpert 2.0.

### 2.4. Design and Assembly of Anti-B7-H3 CARs

**Figure 1:**
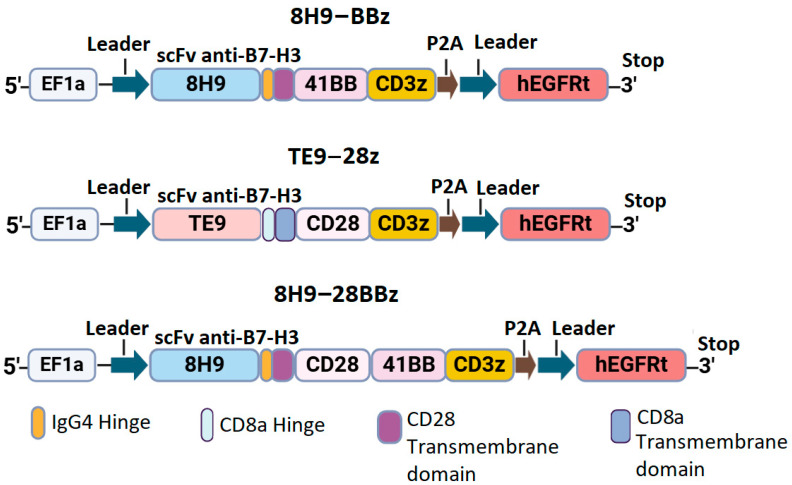
Schematic representation of anti-B7-H3 CAR constructs.

We designed three constructs encoding the variants of CAR with different scFv targeting B7-H3: the second-generation CAR with the 4-1BB costimulatory domain (8H9-BBz), the CAR with dual CD28 and 4-1BB domains (8H9-28BBz), and the second-generation CAR with CD28 co-stimulation (TE9-28z). All constructs included a FLAG tag at the N-terminus, scFv, IgG4/CD8 hinge region, CD8/CD28 transmembrane domain, intracellular costimulatory domains (CD28 and/or 4-1BB), and CD3ζ signaling domain. A truncated EGFRt tag linked with a P2A peptide was used for monitoring CAR-T cell expansion (Figure 1).

Synthetic DNA sequences encoding scFv fragments and other CAR components were obtained from Synbio Technologies. CAR constructs were cloned and inserted into the pWPXL vector (Addgene plasmid #12257) using restriction enzymes BamHI and EcoRI (New England Biolabs, Ipswich, MA, USA). After that, they were transformed into NEB Stable Competent *E. coli* cells. The correct assembly of the constructs was verified via restriction mapping and Sanger sequencing of the plasmid DNA.

### 2.5. Isolation of T Cells

Human PBMCs were isolated from the blood of healthy donors and collected into tubes with EDTA via gradient density centrifugation, following the standard protocol approved by the local ethics committee. All participants provided informed written consent. Mononuclear cells (MNCs) were counted in 3% acetic acid solution using a Goryaev chamber. T cells were isolated from human PBMCs with an EasySep™ Human T Cell Isolation Kit (Thermo Fisher Scientific Inc., Waltham, MA, USA). T cell viability and count were assessed using 0.4% trypan blue dye exclusion in a Goryaev chamber. A CD4/CD8 ratio and its phenotypes were assessed via flow cytometry.

### 2.6. Transduction of T Cells with Pseudoviral Particles

Lentiviral particles containing CARs were produced via the polyethylenimine-mediated co-transfection of HEK293T cells with the corresponding lentiviral CAR plasmids and the packaging plasmids, pCMV dR8.91 and pMD2.G (Addgene plasmid #12259, a gift from Didier Trono). Supernatants were collected 48 h post-transfection and purified via centrifugation (3000× *g*, 18 h, 4 °C), then frozen at −80 °C until use. Freshly selected T-lymphocytes were activated with Dynabeads Human T-Activator CD3/CD28 beads (Thermo Fisher Scientific, Vilnius, Lithuania) at a 1:1 ratio for 48 h in RPMI-1640 medium supplemented with 10% fetal bovine serum (Thermo Fisher Scientific, Vilnius, Lithuania), 1× antibiotic–antimycotic solution, and cytokines IL-7, IL-15, and IL-21 (Miltenyi Biotec, Bergisch Gladbach, Germany) at a concentration of 10 ng/mL. The activated T cells were mixed with 10 MOI of lentiviral supernatants and placed into plates pre-coated with 13 μg/mL RetroNectin (Takara Bio, Goteborg, Sweden). The plates were centrifuged at 600× *g* for 90 min at 32 °C and incubated at 37 °C with 5% CO_2_ for 18 h. A total of 24 h post-transduction, the cells were washed with complete media containing cytokines and further incubated in vitro for 8–12 days. On days 10–12, cell expansion, phenotype, and tEGFR expression were monitored. Non-transduced lymphocytes served as MOCK controls.

### 2.7. NFAT-Inducible Reporter Assay

In activation assays, 50,000 CAR-expressing Jurkat-NFAT-GFP cells were co-incubated for 18 h with 50,000 target cell lines pre-stained with CellTrace Violet (Thermo Thermo Fisher Scientific Inc., Waltham, MA, USA). Their activation was evaluated using the percentage of GFP+ cells (normalized to CAR+ cells) and the mean fluorescence intensity (MFI) of the GFP signal.

### 2.8. Cytotoxicity Assay

The assay was performed in a 96-well plate format by co-incubating effector cells (CAR-T or MOCK control) with target cells for 16 h. Target cells LAN1 and 143B were labeled with CellTrace Violet prior to the assay to distinguish them from effector cells. Triplicate measurements were conducted at three effector-to-target (E:T) ratios: 2:1, 1:1, and 1:2. Cell death was quantified via flow cytometry after staining with 7-AAD, whereas cytotoxic activity (CTA) was calculated using the following formula: (7AAD^+^ with effectors) − (7AAD^+^ w/o effectors)/100% − (7AAD^+^ w/o effectors).

### 2.9. CAR-T Cell Exhaustion Assay with Repeated Stimulation

Effector and target cells were co-cultured in a 96w plate at an E:T ratio of 1:3 on day 0 (3000 CAR-T and 9000 target cells). The assay was carried out in complete RPMI-1640 medium supplemented with 10% FBS, in the absence of exogenous cytokines. LAN1 and 143B cells were pre-stained with CellTrace Violet. Every 2 days, one single well was evaluated for target cell death (7-AAD staining) and CAR-T persistence (EGFRt staining) via flow cytometry. A total of 9000 freshly labeled target cells were added to the remaining wells. On days 0, 2, and 9, CAR-T exhaustion was assessed via staining for TIM-3 (PE), TIGIT (PE-Cy7), and PD-1 (APC-750).

### 2.10. Cytokine Secretion Induction

Prior to the assay, effector cells were washed with fresh culture medium and rested for 24 h without cytokine supplementation. The experiment was conducted in 24-well plates by co-incubating effector cells with WT Jurkat and Jurkat B7-H3-positive cell lines at a 1:1 ratio. After one hour of co-incubation, monensin (BioLegend, San Diego, CA, USA) was added to each well. After fixation and permeabilization, the intracellular staining for IFNγ and TNFα was performed using the eBioscience™ intracellular cytokine staining kit (Life Technologies, Carlsbad, CA USA). Flow cytometric analysis was performed using antibodies against CD3 (FITC), EGFRt (APC), IFNγ (PE), and TNFα (PE-Cy7). As a positive control, mock-transduced T cells were stimulated with phorbol 12-myristate 13-acetate (PMA, 5 ng/mL) and ionomycin (1 μg/mL).

### 2.11. Statistical Analysis

Statistical analysis was performed using the GraphPad Prism version 8.0 and R software 4.3.1 (RStudio). The differences in activation and cytotoxic activity were compared using Student’s *t*-test for independent samples (assuming equal variances) or Welch’s *t*-test (for unequal variances). A *p*-value < 0.05 was considered statistically significant.

## 3. Results

### 3.1. B7-H3 Expression in Neuroblastoma Tumor Samples

**Figure 2:**
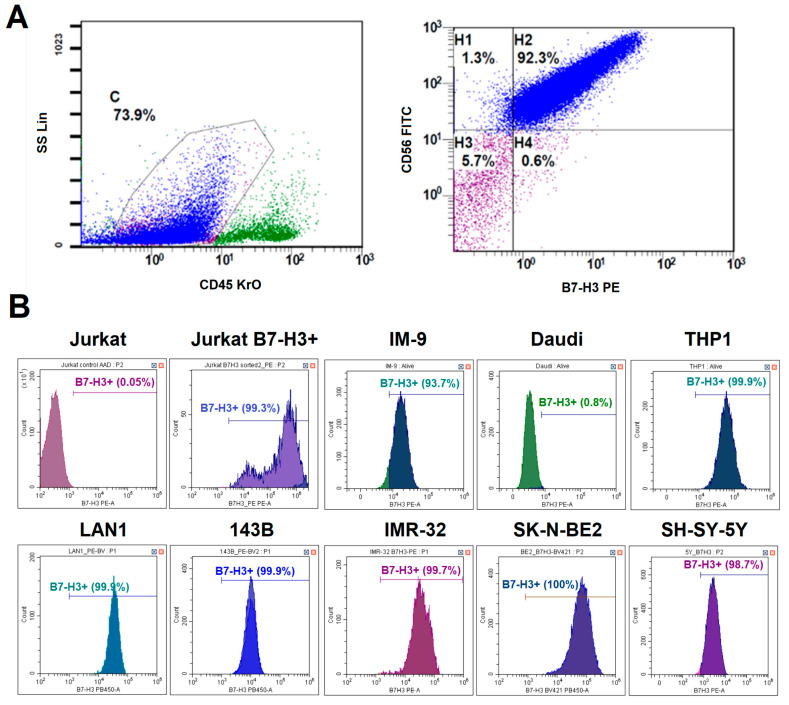
Analysis of B7-H3 expression on different tumor cells. (**A**)—A schematic representation of the gating strategy for the analysis of neuroblastoma patient samples. (**B**)—The histogram plots of B7-H3 expression in different tumor cell lines. Live cells were gated prior to the analysis. The lines show the percentage of B7-H3-positive cells.

Since B7-H3 is often overexpressed in many types of cancers, it is included in many diagnostic panels used for immunophenotyping tumor biopsies. To determine whether B7-H3 could be a promising target for CAR-T cell therapy for neuroblastoma, we evaluated B7-H3 expression in different patients. Tumor biopsies for the suspected primary disease and bone marrow samples with the confirmed neuroblastoma were obtained from 25 patients. The region containing tumor cells was characterized by a CD45^−^, CD56^+^, and CD81^+^ phenotype. The expression of GD2 and B7-H3 was considered positive if it was observed on more than 20% of tumor cells. B7-H3 expression was detected in 23 out of 25 (92%) neuroblastoma cases. All samples with significant tumor infiltration had a typical phenotype: CD45^−^, CD56^+^, CD81^+^, GD2^+^, B7-H3^+^. Two B7-H3- cases were associated with low tumor burden in bone marrow. An example of tumor cell gating is shown in Figure 2A. High expression levels of B7-H3 in neuroblastoma without prominent heterogeneity were confirmed via quantitative PCR in our earlier study [18].

### 3.2. Differential B7-H3 Expression in Model Cell Lines

To select an appropriate tumor model to analyze the relevance of anti-B7-H3 CAR-T cell therapy, we evaluated the expression levels of B7-H3 in a panel of model cell lines representing various malignancies. We evaluated B7-H3 expression across three neuroblastoma cell lines (LAN-1, IMR-32, and SK-N-BE(2)), an osteosarcoma line (143B), a T cell line (Jurkat), B cell lines (Daudi and IM-9), and a myeloid cell line (THP1) via flow cytometry (Figure 2B). All neuroblastoma lines were 100% B7-H3^+^ with high mean fluorescence intensity (ΔMFI = ~2 × 10^5^). Osteosarcoma cell lines and IM-9 were also B7-H3^+^, but with lower expression levels (ΔMFI = ~1 × 10^5^). Jurkat and Daudi cells were negative for B7-H3. Therefore, LAN-1 (high B7-H3), 143B (moderate B7-H3), and Jurkat/Daudi (B7-H3^−^) were selected as target cells for functional assays.

### 3.3. High Activity of TE9-Derived scFv CARs in the NFAT-GFP Reporter Assay

**Figure 3:**
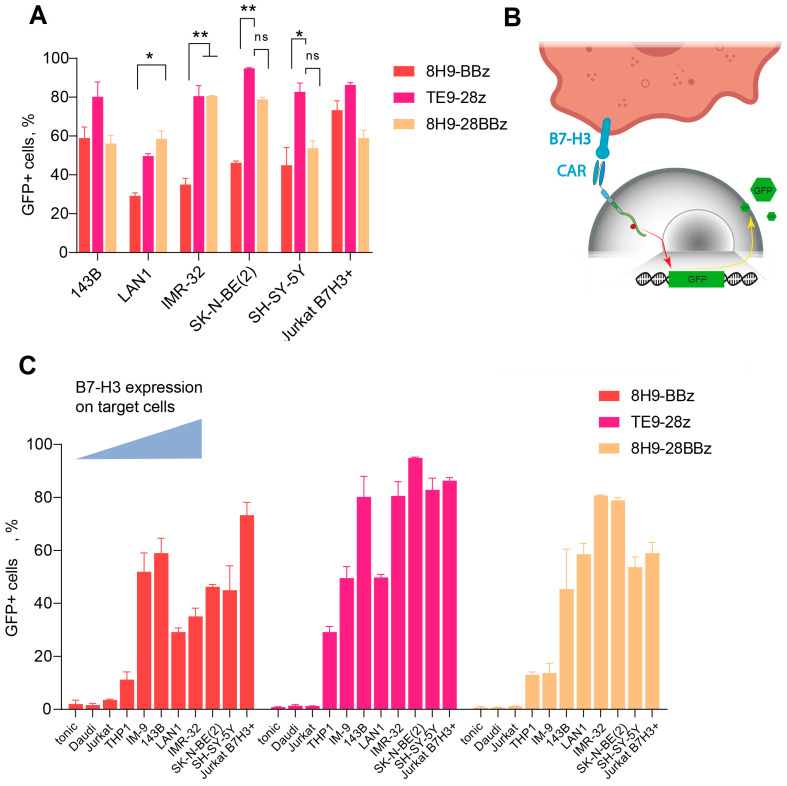
Generation of functional active anti-B7-H3 CAR Jurkat cells. (**A**)—A comparative activation of the three Jurkat-NFAT-GFP CAR variants. The data were adjusted against the level of GFP expression in Mock T cells (tonic signaling) (data = experimental data − tonic signaling). (**B**)—A schematic representation of Jurkat-NFAT-GFP reporter cell workflow. (**C**)—Fluorescence assay on Jurkat-NFAT-GFP cells transduced with 8H9-BBz, TE9-28z, and 8H9-28BBz backbones and activated in the presence of different B7-H3+ target cells with increasing B7-H3 expression. Baseline (tonic) activation and B7-H3-negative Daudi and Jurkat cells were used as a negative control. All data represent the mean ± SD. * indicates *p* ≤ 0.05, ** indicates *p* ≤ 0.01, ns indicates ‘not significant’. The *p* values were determined via multiple unpaired *t*-tests.

To assess the functionality of anti-B7-H3 CAR cells, we designed several second- and third-generation CAR constructs based on TE9 and 8H9 scFv (Figure 1). We inserted a truncated EGFRt tag linked by a P2A peptide for CAR-T cell expansion monitoring. To analyze the antigen-specific activation of the CAR-T cell panel, we generated a GFP reporter system by transducing Jurkat cells with a construct expressing GFP under the NFAT-inducible promoter. As a reporter cell line, we selected a clonal Jurkat-NFAT-GFP cell line lacking background fluorescence in the absence of stimulation (Figure 3B). These reporter cells were subsequently transduced with a panel of different CAR backbones. Background activation in the absence of target cells was low (<10%) across all receptors and comparable to the non-specific signals observed with B7-H3^−^ targets (Daudi, NXS2, Jurkat). Specific activation was measured using GFP upregulation upon co-culture with B7-H3^+^ cells (143B and LAN-1) and correlated with B7-H3 expression levels (Figure 3C). TE9-28z CAR showed significantly higher activation compared to 8H9-BBz when stimulated with neuroblastoma cells (*p* < 0.05 for IMR-32 and SK-N-BE(2)). The activation of the third-generation 8H9-28BBz receptor was higher than its second-generation counterpart, 8H9-BBz, specifically in response to IMR-32 cells. However, this increase in activity did not reach statistical significance when compared to the TE9-28z construct across the panel of tested targets. Notably, TE9-28z consistently demonstrated superior functional activity relative to both 8H9-based receptors, irrespective of the target cell line, suggesting a more potent antitumor response mediated by the TE9-derived scFv (Figure 3A).

### 3.4. Cytokine Secretion by Anti-B7-H3 CAR-T Cells

**Figure 4:**
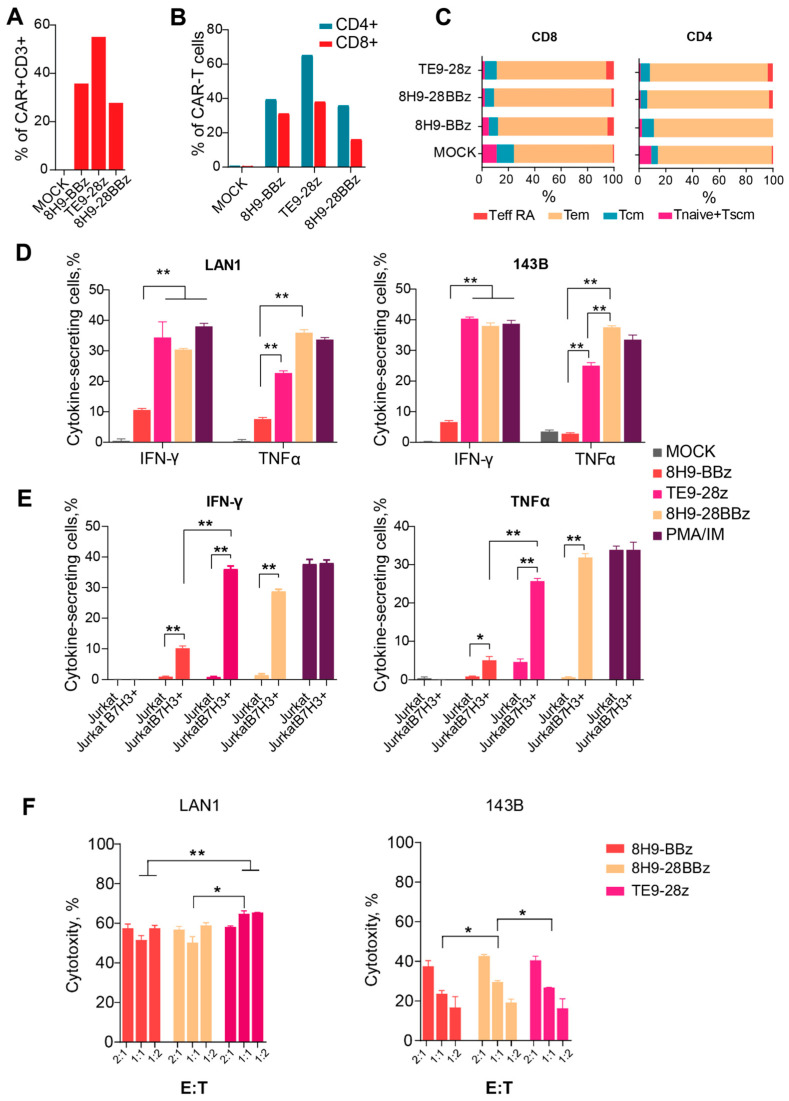
Analysis of the functional activity of anti-B7-H3 CAR T cells. (**A**)—CAR-T cell transduction level. (**B**)—Expression of CAR in CD4+ and CD8+ T cells. (**C**)—The differentiation status of CD4+ and CD8+ of the 8H9-BBz, TE9-BBz, and 8H9-28BBz anti-B7-H3 CAR-T cells. Tem—effector memory T cell, Tcm—T cell central memory, T naive—T cell-naive, T eff RA—terminally differentiated T cells, Tscm—T memory stem cell. (**D**)—Cytokine secretion (IFNγ and TNFα) by anti-B7-H3 CAR-T cells upon stimulation with LAN1 and 143B target cells. (**E**)—Cytokine secretion (IFNγ and TNFα) by anti-B7-H3 CAR-T cells upon stimulation with wild-type (WT) and B7-H3+ Jurkat cells. Mock T cells were used as a negative control. PMA/Ionomycin stimulation was used as a positive control. (**F**)—The comparison of cytotoxic activity of anti-B7-H3 CAR-T cells against two B7-H3-expressing cell lines—LAN-1 (neuroblastoma) and 143B (osteosarcoma)—at varying effector-to-target ratios (E:T—2:1; 1:1; 1:2). The data were adjusted against a negative control (Mock T cells). All data represent the mean ± SD. * indicates *p* ≤ 0.05, ** indicates *p* ≤ 0.01. The *p*-values were determined by multiple unpaired *t*-tests.

To further validate the functional activation of CAR-T cells, we measured IFNγ and TNFα secretion following a 24 h co-culture with target cells at a 1:1 effector-to-target ratio. All CAR variants specifically secreted both cytokines in response to B7-H3-positive Jurkat cells, but not in response to WT Jurkat cells, confirming antigen-specific activation (Figure 4E). Notably, 8H9-BBz CAR-T cells produced significantly lower levels of IFNγ and TNFα compared to both 8H9-28BBz and TE9-28z variants (*p* = 0.04). While IFNγ production was comparable between TE9-28z and 8H9-28BBz, the latter demonstrated superior TNFα secretion, suggesting enhanced functional potency in this context (Figure 4D).

### 3.5. Phenotypic Analysis of Anti-B7-H3 CAR-T Cells

We evaluated the phenotype of the produced anti-B7-H3 CAR-T cells and analyzed CAR expression and differentiation status 12 days post-transduction for each CAR variant via flow cytometry. Untransduced T cells were used as a negative (Mock) control (Figure 4A). In all samples, CD3^+^ T cells comprised the vast majority of the population, consistently exceeding 90% (ranging from 92% to 99%). The CD4/CD8 ratio was approximately 3:2. Transduction efficiency was assessed using the FLAG tag, which ranged from 25% to 65% in CD4^+^ T cells (median: 45%) and from 18% to 40% in CD8^+^ T cells (median: 29%) (Figure 4B).

To determine the differentiation status, we used a panel of surface markers, namely, CD45RO, CCR7, and CD62L, which let us classify T cell subsets into naïve (Tnaiv), stem memory (Tscm), central memory (Tcm), transitional memory (Ttm), and effector (Teff) populations (Figure 4C). The combined proportion of early memory subsets (CCR7^+^ cells: Tnaiv + Tscm + Tcm) was used as a surrogate measure of proliferative potential. In the MOCK-transduced controls, the percentages of CCR7^+^ cells were 22% among CD4^+^ T cells and 21% among CD8^+^ T cells. CAR-modified T cells maintained the comparable proportions of these early memory subsets, with CCR7^+^ frequencies ranging from 22.6% to 31.5% in CD4^+^ cells and from 21.7% to 26.3% in CD8^+^ cells. In summary, the phenotypic analysis demonstrated that the anti-B7-H3 CAR-T cells maintained a favorable differentiation profile comparable to that of the untransduced T cells.

### 3.6. Lysis of Osteosarcoma and Neuroblastoma Cell Lines Induced by Anti-B7-H3 CAR-T Cells In Vitro

Assessing the cytotoxic potential of CAR-T cells is a critical step in evaluating their functional activity and therapeutic relevance. We evaluated the cytotoxic activity of anti-B7-H3 CAR-T cells against two B7-H3-expressing cell lines, LAN-1 (neuroblastoma) and 143B (osteosarcoma), at varying effector-to-target ratios. (Figure 4F). All CAR variants exhibited dose-dependent cytotoxic activity against B7-H3^+^ lines. The CTA of the third-generation receptor 8H9-28BBz slightly exceeded the CAT of the 8H9-BBz receptor with 143B targets (*p* = 0.03 for E/T = 1:1); however, we observed no differences in the case of LAN1 targets. When comparing the CTA of the 8H9-BBz and TE9-28z receptors, the latter performs better with LAN1 targets (*p* < 0.001 for E/T = 1:1). Interestingly, all CAR-T cells exhibited significantly higher cytotoxicity against the neuroblastoma-derived LAN-1 cells compared to the osteosarcoma-derived 143B line, with up to a 1.5- to 3-fold increase in CTA at E/T = 1:1 (*p* < 0.05.), likely arising from the possible resistance of osteosarcoma cells to lymphocyte-mediated killing.

### 3.7. Expansion Dynamics and Exhaustion Profiles of Anti-B7-H3 CAR-T Cells in a Sequential Killing Assay

**Figure 5:**
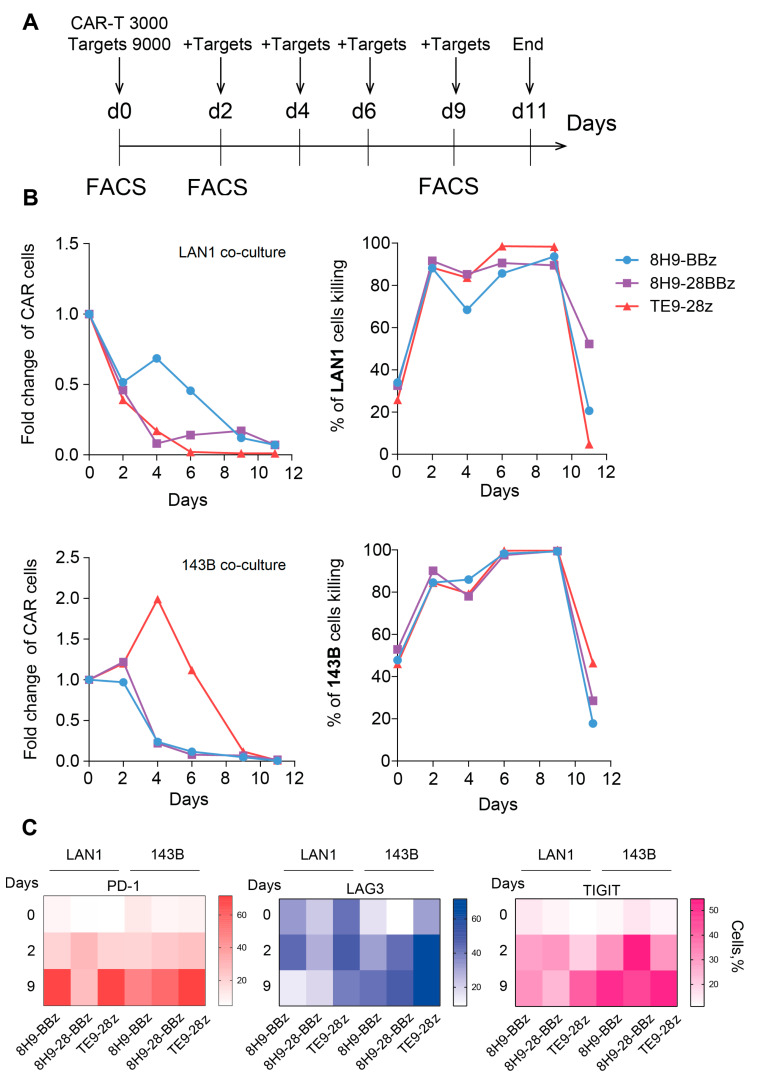
Sequential killing assay and exhaustion analysis of anti-B7-H3 CAR T cells. (**A**)—Experimental timeline. CAR-T cells were co-cultured with B7-H3-positive target cells (LAN-1 or 143B) in a sequential killing assay. Fresh target cells were added every 2 days to maintain continuous antigenic stimulation. On days 0, 2, and 9 of the experiment, CAR-T cells were analyzed via flow cytometry for the expression of exhaustion markers (LAG-3, TIGIT, PD-1). (**B**)—Sequential killing assay on CAR-T cells against two B7-H3-expressing cell lines—LAN-1 (neuroblastoma) and 143B (osteosarcoma). The absolute number of live CAR-T cells (left panel) and live target cells (right panel) was monitored over time during repeated stimulation. (**C**)—Heatmap of exhaustion marker expression (LAG-3, TIGIT, PD-1) on anti-B7-H3 CAR-T cells on days 0, 2, and 9 of the sequential killing assay with LAN1 and 143B target cells. The color represents the number of marker-positive cells (%).

To assess the functional durability and therapeutic potential of anti-B7-H3 CAR-T cells, we evaluated their persistence, ability to control tumor outgrowth, and development of exhaustion phenotypes over an 11-day period in vitro. This ‘sequential killing’ assay was designed to observe the long-term cytotoxic activity and replicative capacity, as well as detect a possible functional decline during a consistent re-challenge of CAR-T cells with fresh portions of target cells (Figure 5A). Under continuous co-culture conditions, all CAR variants efficiently control target cells, resulting in more than 80% death of target cells in the period from day 2 to 9, with a maximum killing on day 9. All three receptors demonstrated almost identical cytotoxic activity, with a slight lag for receptor 8H9-BBz with target cells LAN1. However, the proliferative capacity and persistence of CAR-T cells varied depending on the CAR structure. Actual expansion was observed only for the TE9-28z receptor with 143B target cells, with maximum growth on day 4. With LAN1 target cells, there was no expansion of any of the three CAR-T variants, but the 8H9-BBz variant showed the best persistence with some improvement on day 4. The experiment was completed on day 11, when only a small number of CAR-T cells remained in the test, which had completely lost their cytotoxic activity (Figure 5B).

To further evaluate functional impairment during prolonged antigen exposure, we analyzed the expression of key exhaustion markers LAG-3, TIGIT, and PD-1 prior to co-culture (day 0) and on days 2 and 6 of culture with B7-H3-expressing targets. In co-cultures with LAN-1 cells, LAG-3 upregulation was detected only at later time points and was elevated on day 9. TIGIT expression increased gradually over time, most notably in the TE9-28z-transduced T cells. PD-1 levels rose significantly by day 12 in both 8H9-BBz and TE9-28z, while remaining relatively stable in 8H9-28BBz. In contrast, in co-cultures with 143B cells, all exhaustion markers continuously increased, irrespective of the CAR design throughout the entire observation period, suggesting a more pronounced T cell exhaustion in response to this tumor type (Figure 5C). Overall, CAR-T cell expansion and exhaustion profiles varied depending on the receptor design and tumor cell line. 8H9-BBz demonstrated the highest persistence in LAN-1 co-cultures, while TE9-28z induced superior expansion with 143B. All variants exhibited the signs of functional exhaustion over time, particularly in response to osteosarcoma cells.

## 4. Discussion

In this study, we compared the functional activity of CAR-T cells containing two of the most promising antigen-binding antibody domains: 8H9 and TE9. The 8H9-based CAR backbones represent both second-generation (with a 4-1BB costimulatory domain) and third-generation CARs (with combined CD28 and 4-1BB domains), while TE9 was considered a second-generation CAR, with a CD28 costimulatory domain.

Murine (chimeric) monoclonal antibody 8H9, originally described as a targeted antibody for a broad spectrum of solid tumors, has been used in the radiolabeled immunotoxin 131I-Omburtamab [19,20]. Several preclinical studies have demonstrated the functional activity of the 8H9 single-chain variable fragment (scFv) in CAR constructs targeting prostate cancer [15], glioblastoma [14], and non-small cell lung cancer [16]. The first phase 1 clinical trial of B7-H3-targeting CAR T cells for diffuse intrinsic pontine glioma, published in 2025, used second-generation CAR MGA271-BBz [12]. In contrast, the novel murine antibody TE9, described by Birley et al., was shown to outperform other anti-B7-H3 antibodies such as MGA271 and 376.96 when expressed in a second-generation CAR format (TE9-28z) [17]. Based on these data, it is interesting to compare the functional activity of second- and third-generation receptors based on the known antibody 8H9 with that of the new receptor variant TE9-28z. A direct comparison between CARs based on 8H9 and TE9 has not previously been reported, making this research a relevant task.

The antigen-specific effector functions of CAR-T cells are largely determined by three key factors: the binding affinity of the antigen-recognition domain, the density of antigen expression on the surface of target cells, and the choice of the intracellular co-stimulatory domain (such as 4-1BB or CD28) introduced into the receptor. Together, these elements affect the strength and duration of T cell activation, as well as the overall functional potency of the engineered cells. Among these, the affinity of the antigen-binding domain plays a critical role in determining the sensitivity and specificity of CAR-T cell engagement with tumor cells. In this regard, the TE9 scFv demonstrates a markedly higher binding affinity to B7-H3 (KD = 0.44 nM) [21], compared to 8H9 (KD = 10 nM) [22]. This difference suggests that TE9-based CARs may be capable of recognizing and activating T cells in response to the lower levels of B7-H3 expression, potentially expanding their therapeutic applicability for tumors with heterogeneous antigen presentation.

In our NFAT reporter activation assay, GFP expression is induced by signaling through the CD3ζ chain upon CAR engagement with an antigen, with little contribution from the costimulatory domains. As expected, the activation levels correlated with B7-H3 surface density: high-expressing cell lines (SK-N-BE(2), SH-SY-5Y, IMR-32, and B7-H3-transduced Jurkat) triggered stronger activation than low-expressing (IM-9, THP-1) or B7-H3-negative cells (Daudi, wild-type Jurkat). Among the three tested CAR variants, TE9-28z demonstrated the highest activation, likely due to its higher antigen affinity. Of the two 8H9-based receptors, the third-generation construct (8H9-28BBz) showed a trend toward increased activation, suggesting that the presence of the CD28-signaling domain may enhance early T cell stimulation.

The cytotoxic activity of all CAR variants was confirmed against LAN-1 (neuroblastoma) and 143B (osteosarcoma) target cells. However, a significantly greater cytotoxicity was observed against LAN-1 compared to 143B cells across all CAR designs. This difference may be attributed to higher B7-H3 expression levels on LAN-1 cells, as well as other mechanisms influencing tumor cell sensitivity to CAR-T. No significant differences in cytotoxic activity were observed between the selected CAR constructs, either in short-term assays or in long-term co-cultures over a 12-day period.

To evaluate CAR-T persistence and exhaustion, we conducted a series of extended co-culture experiments with repeated restimulation using LAN-1 and 143B target cells. This model most accurately demonstrated the ability of a limited number of CAR-T cells to control a large tumor burden while maintaining proliferative capacity. Long-term monitoring revealed better persistence of the 8H9-BBz CAR-T cells between days 2 and 6 in co-culture with LAN-1 cells, as well as more pronounced expansion of TE9-28z CAR-T cells in the case of 143B cells. The expansion of TE9-28z CAR-T with 143B cells is entirely consistent with the results of the NFAT reporter activation assay, where TE9-28z CAR-T activation was greatest on 143B targets. Interestingly, the same TE9-28z CAR-T behaves completely differently in co-culture with 143B and LAN1 target cells. With the latter, the level of TE9-28z CAR-T rapidly declined while maintaining high cytotoxic activity, which is presumably associated with activation-induced cell death. In contrast, the 8H9-BBz CAR-T variant with LAN1 target cells maintained the best persistence, without significant expansion, but was inferior to other CAR-Ts in terms of cytotoxic activity, which was noticeable on days 4–6. These data can be interpreted as insufficient activation of this receptor, which is consistent with the low activation of the Jurkat NFAT reporter 8H9-BBz with LAN1 targets (Figure 3C).

The analysis of exhaustion markers (LAG-3, TIGIT, PD-1) on days 2 and 9 yielded ambiguous results, with an overall increase in the expression of exhaustion markers during the test. The expression of exhaustion markers on day 0 corresponds to the resting state of cells growing prior to the start of the assay in the presence of cytokines but without antigen stimulation. On day 0, PD-1 and TIGIT expression are virtually absent, but basal TIM-3 expression is observed, which is minimal in 8H9-28BBz CAR-T. PD-1 expression significantly increased by day 12 in the case of 8H9-BBz and TE9-28z but remained stable for 8H9-28BBz between days 9 and 12. LAG-3 expression did not increase significantly in the cultures with LAN-1 cells but rose notably in all three CAR variants during the co-culture with 143B cells. A more pronounced increase in TIGIT was also observed in the 143B co-cultures. Importantly, no statistically significant differences in marker expression were observed among (across) the different CAR designs. This suggests that the observed variations in functional activity likely do not depend on the differences in exhaustion marker induction. However, the interpretation of these findings remains multifaceted. On the one hand, upregulated exhaustion markers can be considered a sign of T cell activation, often correlating with ongoing effector function. On the other hand, persistent expression of these markers is associated with functional impairment and eventual apoptosis of T cells. In the case of comparable activation and cytotoxicity levels, CAR-T cells that exhibit lower levels of exhaustion markers may have a survival advantage, potentially sustaining long-term anti-tumor activity. This hypothesis is also supported by the recent findings of Harush et al. [23]. The expression of these markers alone does not definitively indicate functional exhaustion, but we can confirm CAR-T cell exhaustion after 9 days of the assay due to the loss of cytotoxic capacity and inability to further control targets. Some methods of CAR-T cell engineering have been developed to overcome the functional exhaustion of CAR-T cells during prolonged exposure to large numbers of tumor cells. This includes the co-expression of the *Δ133p53a* gene isoform [24] and the c-jun transcription factor [25], as well as the knockout of the *BATF* gene [26].

Among the assays, cytokine secretion following co-culture proved to be the most sensitive method for estimating functional CAR-T cell activity. All variants induced antigen-specific IFNγ and TNFα production; however, we observed notable differences between the constructs. Both 8H9-28BBz and TE9-28z triggered more pronounced cytokine responses compared to 8H9-BBz, which confirms their superior functional potency. TE9-28z showed a trend toward higher IFNγ secretion, while 8H9-28BBz demonstrated enhanced TNFα production. These distinct cytokine profiles might indicate differences in co-stimulatory signaling and contribute to the in vivo antitumor response and immunomodulatory effects.

In summary, all three CAR variants demonstrated the specific recognition of B7-H3-expressing cells and exhibited comparable in vitro cytotoxic activity against neuroblastoma and osteosarcoma cell lines. However, their functional activation and cytokine production differed significantly. Both 8H9-28BBz and TE9-28z outperformed the second-generation 8H9-BBz receptor, exhibiting enhanced activation profiles and more robust cytokine responses. Our findings suggest that employing multiple costimulatory domains, as seen in the third-generation 8H9-28BBz and high-affinity scFv such as TE9, can significantly improve CAR-T cell efficiency. Taking into account the limitations associated with the in vitro results, we can rationalize the selection of 8H9-28BBz and TE9-28z for further preclinical evaluation in in vivo tumor models to assess their therapeutic potential and possible transition to clinical use.

## Data Availability

The main data supporting the results in this study are available within the paper and the Supplementary Materials. All data generated in this study can be obtained from the corresponding authors upon reasonable request.

## Supplementary Materials

The following supporting information can be downloaded at https://www.mdpi.com/article/10.3390/biomedicines13092130/s1: Table S1. List of antibodies used for flow cytometry. Table S2. List of antibodies used for flow cytometry.
