# Correction: Lutskovich et al. Comparative Preclinical Analysis of Anti-B7-H3 CAR-T Cells Targeting Neuroblastoma. *Biomedicines* 2025, *13*, 2130

**DOI:** 10.3390/biomedicines14051059

**Published:** 2026-05-07

**Authors:** Dzmitry V. Lutskovich, Alexander N. Meleshko, Valeria M. Stepanova, Dmitri O. Dormeshkin, Yury P. Rubtsov

**Affiliations:** 1 Belarusian Research Center for Pediatric Oncology, Hematology and Immunology, 223053 Minsk, Belarus; lutskovichdm@gmail.com; 2 Shemyakin-Ovchinnikov Institute, Bioorganic Chemistry of the Russian Academy of Sciences, Moscow 117997, Russia; ukrainskaya49@gmail.com (V.M.S.); yrubtsov@ibch.ru (Y.P.R.); 3 Institute of Bioorganic Chemistry of the National Academy of Sciences of Belarus, 220141 Minsk, Belarus; dormeshkin@gmail.com

## Error in Spelling

In the original publication [1], ‘TE9-28z’ was misspelled as ‘TE9-BBz’. ‘TE9-BBz’ shall be replaced with ‘TE9-28z’ in the main text (Section 3.6, Figure 3C caption and Figure 5C). The corrected Paragraph, Figure 3C caption and Figure 5C appear below.

Assessing the cytotoxic potential of CAR-T cells is a critical step in evaluating their functional activity and therapeutic relevance. We evaluated the cytotoxic activity of anti-B7-H3 CAR-T cells against two B7-H3-expressing cell lines, LAN-1 (neuroblastoma) and 143B (osteosarcoma), at varying effector-to-target ratios. (Figure 4F). All CAR variants exhibited dose-dependent cytotoxic activity against B7-H3^+^ lines. The CTA of the third-generation receptor 8H9-28BBz slightly exceeded the CAT of the 8H9-BBz receptor with 143B targets (*p* = 0.03 for E/T = 1:1); however, we observed no differences in the case of LAN1 targets. When comparing the CTA of the 8H9-BBz and TE9-28z receptors, the latter performs better with LAN1 targets (*p* < 0.001 for E/T = 1:1). Interestingly, all CAR-T cells exhibited significantly higher cytotoxicity against the neuroblastoma-derived LAN-1 cells compared to the osteosarcoma-derived 143B line, with up to a 1.5- to 3-fold increase in CTA at E/T = 1:1 (*p* < 0.05.), likely arising from the possible resistance of osteosarcoma cells to lymphocyte-mediated killing.

**Figure 3.** Generation of functional active anti-B7-H3 CAR Jurkat cells. (**A**)—A comparative activation of the three Jurkat-NFAT-GFP CAR variants. The data were adjusted against the level of GFP expression in Mock T cells (tonic signaling) (data = experimental data − tonic signaling). (**B**)—A schematic representation of Jurkat-NFAT-GFP reporter cell workflow. (**C**)—Fluorescence assay on Jurkat-NFAT-GFP cells transduced with 8H9-BBz, TE9-28z, and 8H9-28BBz backbones and activated in the presence of different B7-H3+ target cells with increasing B7-H3 expression. Baseline (tonic) activation and B7-H3-negative Daudi and Jurkat cells were used as a negative control. All data represent the mean ± SD. * indicates *p* ≤ 0.05, ** indicates *p* ≤ 0.01, ns indicates ‘not significant’. The *p* values were determined via multiple unpaired *t*-tests.

**Figure 5:**
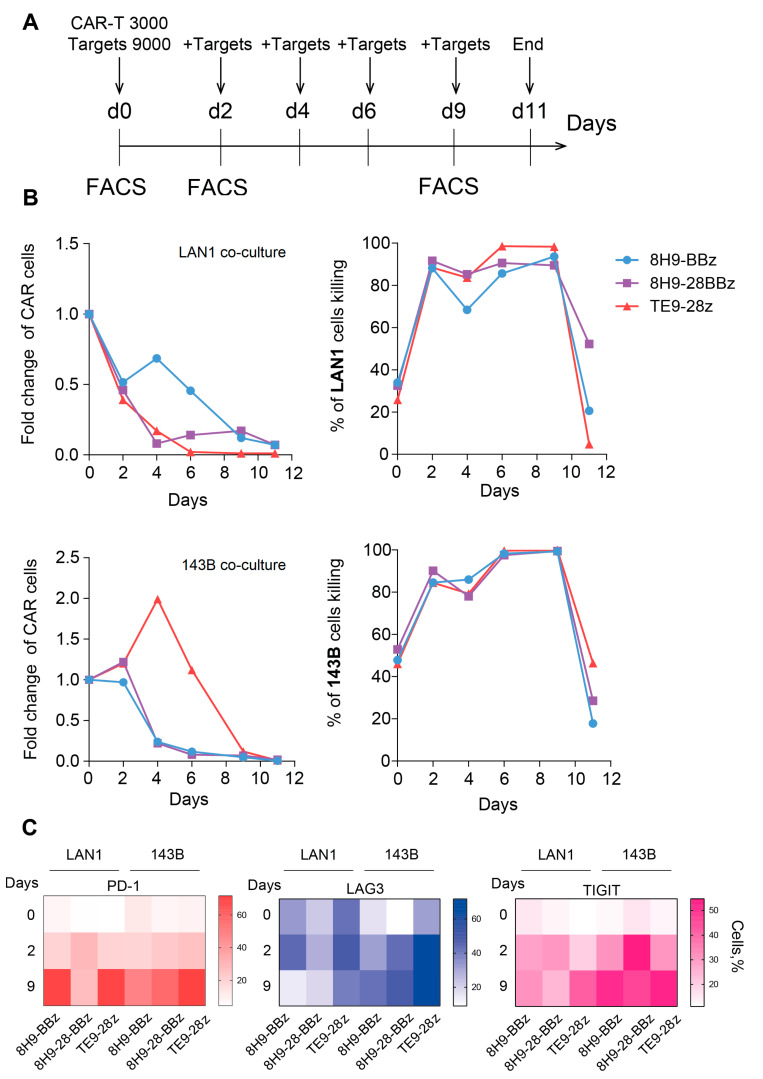
Sequential killing assay and exhaustion analysis of anti-B7-H3 CAR T cells. (**A**)—Experimental timeline. CAR-T cells were co-cultured with B7-H3-positive target cells (LAN-1 or 143B) in a sequential killing assay. Fresh target cells were added every 2 days to maintain continuous antigenic stimulation. On days 0, 2, and 9 of the experiment, CAR-T cells were analyzed via flow cytometry for the expression of exhaustion markers (LAG-3, TIGIT, PD-1). (**B**)—Sequential killing assay on CAR-T cells against two B7-H3-expressing cell lines—LAN-1 (neuroblastoma) and 143B (osteosarcoma). The absolute number of live CAR-T cells (left panel) and live target cells (right panel) was monitored over time during repeated stimulation. (**C**)—Heatmap of exhaustion marker expression (LAG-3, TIGIT, PD-1) on anti-B7-H3 CAR-T cells on days 0, 2, and 9 of the sequential killing assay with LAN1 and 143B target cells. The color represents the number of marker-positive cells (%).

## Error in Figure

In the original publication [1], there was a mistake in Figure 4F as published. The bar chart in the original Figure 4F contained extra cyan-blue bars corresponding to a receptor design not described in this paper. This cyan bar has been removed in the updated figure. The corrected Figure 4F appears below. The authors state that the scientific conclusions are unaffected. This correction was approved by the Academic Editor. The original publication has also been updated.

**Figure 4:**
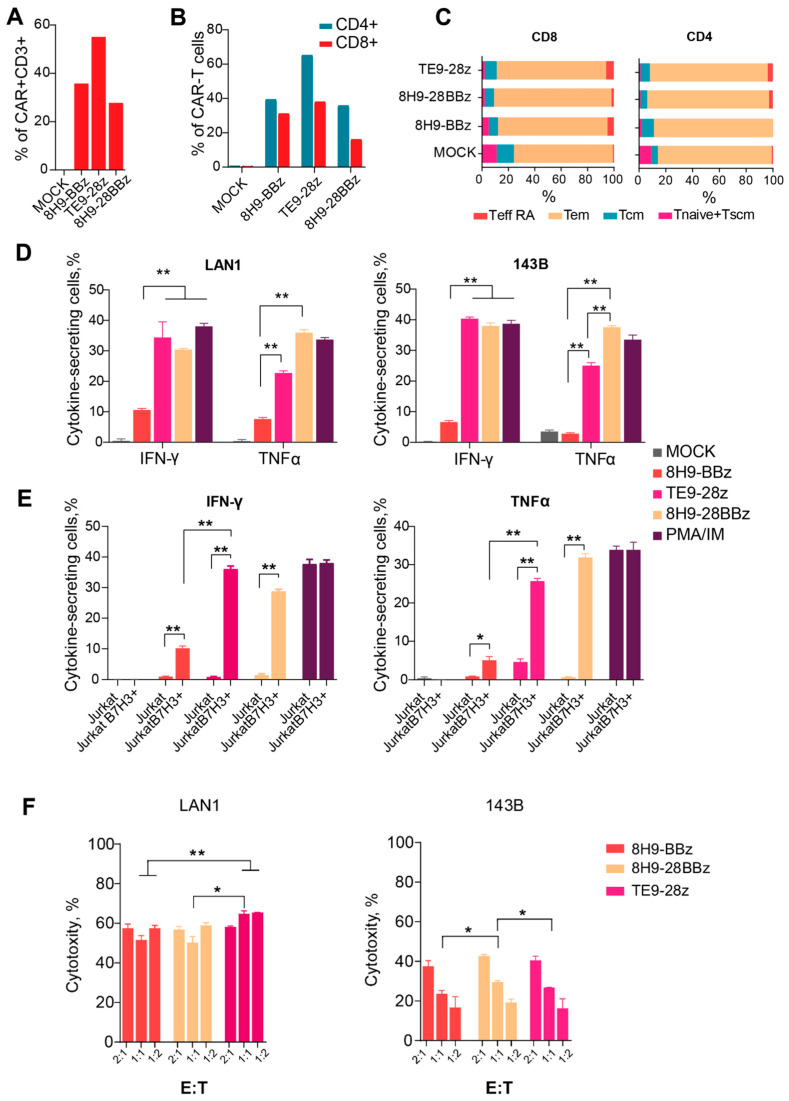
Analysis of the functional activity of anti-B7-H3 CAR T cells. (**A**)—CAR-T cell transduction level. (**B**)—Expression of CAR in CD4+ and CD8+ T cells. (**C**)—The differentiation status of CD4+ and CD8+ of the 8H9-BBz, TE9-BBz, and 8H9-28BBz anti-B7-H3 CAR-T cells. Tem—effector memory T cell, Tcm—T cell central memory, T naive—T cell-naive, T eff RA—terminally differentiated T cells, Tscm—T memory stem cell. (**D**)—Cytokine secretion (IFNγ and TNFα) by anti-B7-H3 CAR-T cells upon stimulation with LAN1 and 143B target cells. (**E**)—Cytokine secretion (IFNγ and TNFα) by anti-B7-H3 CAR-T cells upon stimulation with wild-type (WT) and B7-H3+ Jurkat cells. Mock T cells were used as a negative control. PMA/Ionomycin stimulation was used as a positive control. (**F**)—The comparison of cytotoxic activity of anti-B7-H3 CAR-T cells against two B7-H3-expressing cell lines—LAN-1 (neuroblastoma) and 143B (osteosarcoma)—at varying effector-to-target ratios (E:T—2:1; 1:1; 1:2). The data were adjusted against a negative control (Mock T cells). All data represent the mean ± SD. * indicates *p* ≤ 0.05, ** indicates *p* ≤ 0.01. The *p*-values were determined by multiple unpaired *t*-tests.

## References

[1] Lutskovich D.V., Meleshko A.N., Stepanova V.M., Dormeshkin D.O., Rubtsov Y.P. (2025). Comparative Preclinical Analysis of Anti-B7-H3 CAR-T Cells Targeting Neuroblastoma. Biomedicines.

