# Supplementary material for: Comparative Preclinical Analysis of Anti-B7-H3 CAR-T Cells Targeting Neuroblastoma
Source: Biomedicines. 2025 Aug 31;13(9):2130. doi: 10.3390/biomedicines13092130 (PMC12467967; doi:10.3390/biomedicines13092130)
Supplement: Supplementary file 1 [file biomedicines-13-02130-s001.zip › biomedicines-3796966-Supplement Table S1 - cell lines.pdf]

Supplement Table S1. The origin of the cell lines used in the study.

**Cell line**	**Origin**
Jurkat	Were purchased from DSMZ Catalog #ACC 282
Jurkat-B7-H3+	Obtained in this study
Daudi	The cells were obtained from the vertebrate cell collection of the Academy of Sciences of Belarus.
IM-9	The cells were obtained from the vertebrate cell collection of the Academy of Sciences of Belarus.
THP1	The cells were obtained from the vertebrate cell collection of the Academy of Sciences of Belarus.
HEK293T	The cells were obtained from the vertebrate cell collection of the Academy of Sciences of Belarus.
IMR-32	The cells were obtained from the vertebrate cell collection of the Academy of Sciences of Belarus.
LAN1	The cells were obtained from the vertebrate cell collection Shemyakin-Ovchinnikov Institute of Bioorganic Chemistry of the Russian Academy of Sciences
SH-SY-5Y	Were purchased from Sputnik: CLS Cell Lines Service 300154
143B	The cells were obtained from the vertebrate cell collection Shemyakin-Ovchinnikov Institute of Bioorganic Chemistry of the Russian Academy of Sciences
KHOS-240S	The cells were obtained from the vertebrate cell collection Shemyakin-Ovchinnikov Institute of Bioorganic Chemistry of the Russian Academy of Sciences
SK-N-BE(2)	The cells were obtained from the vertebrate cell collection Shemyakin-Ovchinnikov Institute of Bioorganic Chemistry of the Russian Academy of Sciences
NXS2	Provided by Prof. Dr. med, PhD Holder N. Lode (Germany)
