# Supplementary material for: Comparative Preclinical Analysis of Anti-B7-H3 CAR-T Cells Targeting Neuroblastoma
Source: Biomedicines. 2025 Aug 31;13(9):2130. doi: 10.3390/biomedicines13092130 (PMC12467967; doi:10.3390/biomedicines13092130)
Supplement: Supplementary file 1 [file biomedicines-13-02130-s001.zip › biomedicines-3796966-Supplement Table S2 - antibodies.pdf]

Supplement Table S2. List of antibodies used for flow cytometry.

CD45 (KrO)	Beckman Culter	J33
CD56 (FITC)	Beckman Culter	N901
CD276 (PE)	BioLegend	DCN.70
GD2 (PE)	BioLegend	IgG2a
CD81 (APC)	Beckman Culter	JS64
CD99 (FITC)	BioLegend	Hec2
CD171 (PE)	R&D Systems	555
CD276 (BV421)	DB Biosciense	7-517
TIM-3 (PE)	Miltenyi Biotec	REA635
TIGIT (PE-Cy7)	Miltenyi Biotec	REA1004
PD-1 (APC-750)	Miltenyi Biotec	REA1165
CD3 (FITC)	Beckman Culter	UCHTI
EGFRt (APC)	Invitrogen	Me183
IFNγ (PE)	Invitrogen	4S.B3
TNFα (PE-Cy7)	BioLegend	MAb11
CD45RA	Invitrogen	HI100
CCR7 (APC)	Miltenyi Biotec	REA546
CD62L	Invitrogen	MEL-14
CD4 (FITC)	Invitrogen	RPA-T4
CD8 (PE)	Invitrogen	RPA-T8
FLAG (PE)	BioLegend	L5
